# Intervention of Fish (*Perca fluviatilis*) Maw Hydrolysate in Cyclophosphamide-Induced Immunosuppressed Mice via NF-κB Pathway

**DOI:** 10.3390/foods15071227

**Published:** 2026-04-03

**Authors:** Jie Song, Zi-Wei Zhao, Qing-Tao Zhan, Xue-Mei Ge, Wen-Sen Liu, Mei-Zhen Peng, Xue Tang, Hui-Ping Liu, Xiang-Rong Cheng

**Affiliations:** 1 School of Food Science and Technology, Jiangnan University, Wuxi 214122, China; 6230111090@stu.jiangnan.edu.cn (J.S.); 13783387551@163.com (Z.-W.Z.); zhanqingtao777@gmail.com (Q.-T.Z.); tangxue@jiangnan.edu.cn (X.T.); 2 State Key Laboratory of Food Science and Resources, Jiangnan University, Wuxi 214122, China; 3 College of Light Industry & Food Engineering, Nanjing Forestry University, Nanjing 210037, China; gexuemei@njfu.edu.cn; 4 Guangdong Food Nutrition (Era Biotech) Engineering Technology Research Center, Shenzhen 518100, China; wsenliu@163.com (W.-S.L.); mzhenp@163.com (M.-Z.P.)

**Keywords:** fish maw, hydrolysate, immunosuppression, cyclophosphamide, transcriptome, NF-κB pathway

## Abstract

Immune dysregulation is a critical driver of various pathological processes. Fish maw (FM) serves as a traditional immunomodulatory food. However, the immunomodulatory properties and mechanisms of fish maw hydrolysate (FMH) remain unclear. Here, low-molecular-weight FMH was prepared from *Perca fluviatilis*, exhibiting a major molecular weight distribution of 73–580 Da (83.89%), enriched in charged and hydrophobic amino acids (28.61% and 67.33%, respectively). Moreover, high-resolution mass spectrometry (HRMS) analysis identified 5 small peptides, including Asp-Leu and Gly-Pro-Ala, alongside 7 collagen-derived polypeptides with characteristic Gly-X-Y repetitive motifs. In cyclophosphamide (CTX)-induced immunosuppressed C57BL/6J mice, FMH significantly ameliorated alterations in peripheral blood cell parameters, regulated cytokine homeostasis, attenuated splenic histopathological lesions, and enhanced splenic lymphocyte proliferation. Mechanistically, thymic transcriptomic profiling identified 2237 DEGs in the CTX vs. CON comparison and 212 DEGs in the CTX+FMH vs. CTX comparison, with the NF-κB signaling pathway significantly enriched. Furthermore, qRT-PCR validated the expression of key NF-κB-related genes, including *IκBα*, *P50*, *P65*, *CHUK*, *IL-1β*, and *IL-6*, while immunohistochemical analysis confirmed reduced PI3K and P65 expression, thereby partly restoring immune homeostasis. These findings support FMH as a potential dietary immunomodulator.

## 1. Introduction

Immunocompromised status may impair host defense and increase susceptibility to disease [1]. Notably, accumulating evidence indicates that immune dysregulation contributes to the pathogenesis of a wide spectrum of diseases, including autoimmune disorders, chronic inflammatory conditions, malignancies, and neurodegenerative diseases [2,3,4,5]. Therapeutic enhancement of host defense responses through immunomodulators has been widely explored as a strategy for improving disease resistance [6]. However, conventional immunomodulators, such as corticosteroids and cyclosporine, may have limitations during long-term use, and prolonged use of some agents may impair immune function or cause other adverse effects [7,8]. Consequently, the development of novel dietary immunomodulatory ingredients with good biocompatibility and bioactivity is of considerable significance.

Fish maw, the dried product derived from swim bladders, contains abundant type I collagen and functional amino acids while remaining low in fat, making it a high-protein and low-fat nutritional resource [9]. Current processing of fish maw mainly relies on conventional cooking methods, which are time-consuming and may cause nutrient loss. Moreover, the bioefficacy of native collagen may be limited by its relatively low absorption efficiency [10]. Enzymatic hydrolysis has recently enabled the production of low-molecular-weight hydrolysates and derived peptides [11]. These bioactive products exhibit enhanced absorption and utilization efficiency [12]. Dai et al. revealed the potential of swim bladder-derived peptides in preventing ulcerative colitis [13]. Li et al. demonstrated that enzymatic hydrolysates from *Gadus morhua* swim bladder possess notable antioxidant and anti-aging properties [14]. Cai et al. identified antioxidant peptides from *Miichthys miiuy* swim bladder, and FPYLRH significantly alleviated H_2_O_2_-induced oxidative damage in human umbilical vein endothelial cells (HUVECs) [15]. Nevertheless, existing research predominantly focuses on anti-inflammatory, antioxidant, and anti-aging activities of fish maw hydrolysates and derived peptides, while their immunomodulatory mechanisms remain insufficiently explored.

Furthermore, current commercial fish maw resources predominantly originate from marine species such as yellow croaker, brown croaker, and codfish, whereas the utilization of freshwater fish maw remains relatively limited [16]. *Perca fluviatilis* (Eurasian perch), a freshwater species widely distributed across Eurasia, has attracted attention because of its rapid growth, disease resistance, and favorable edible quality [17]. The feasibility and mechanism of fish maw hydrolysate derived from *Perca fluviatilis* as an immunomodulatory ingredient deserve further investigation. Therefore, to diversify the raw material sources and processing strategies for fish maw while exploring its potential in immune restoration, FMH was prepared from *Perca fluviatilis* maw and systematically evaluated in a CTX-induced immunosuppressed mouse model. CTX is widely used to establish a robust and reproducible immunosuppressed mouse model by inducing myelosuppression and systemic immune dysfunction, and it is therefore suitable for evaluating immunomodulatory interventions in vivo [18,19]. This study aimed to investigate the immunomodulatory efficacy and mechanism of FMH and to provide a theoretical basis for developing dietary immunomodulators.

## 2. Materials and Methods

### 2.1. Materials and Reagents

The fish maw was purchased from Sanhai Industrial Co., Ltd., Guangdong, China. CTX (PHR1404) was purchased from Sigma Aldrich, Shanghai, China. Alcalase 2.4 L (2.4 AU-A/g) was purchased from Novozymes (China) Investment Co., Ltd., Beijing, China. Interleukin-3 (IL-3), interleukin-6 (IL-6), and tumor necrosis factor alpha (TNF-α) assay kits were purchased from Xiamen Huijia Biotechnology Co., Ltd., Xiamen, China. The target gene primers were purchased from Sangon Biotech Co., Ltd., Shanghai, China.

### 2.2. Preparation of the FMH

The fish maw (FM) was initially purified through a series of steps to remove impurities and enhance its quality. At the beginning, degreasing was performed by soaking the FM in petroleum ether for 24 h to eliminate lipid content, using a solid-to-solvent ratio of 1:6 (*w*/*v*). Subsequently, decalcification was carried out using a 0.8 M HCl solution for 12 h to remove calcium deposits, using a solid-to-solvent ratio of 1:10 (*w*/*v*), followed by salting out with a Na_2_SO_4_ solution for an additional 12 h. The purified FM was then dried in a forced air drying oven at 50 °C until constant weight. The dried FM was placed in sealed plastic bags and stored at −20 °C until use. For enzymatic hydrolysis, the dried FM was rehydrated by soaking in purified water (1:20, *w*/*v*) for 12 h. The hydrated FM was boiled for 20 min to soften its structure and then homogenized using a blender at low speed for 30 s followed by high speed for 30 s to obtain a slurry. The pH of the homogenate was adjusted to 9.0 using 0.5 M NaOH or 0.5 M HCl, and alcalase 2.4 L was added at 200 μL/g FM to initiate hydrolysis. The mixture was incubated at 50 °C for 6 h to optimize peptide release, as previously reported for similar collagen hydrolysis processes [20]. After hydrolysis, the reaction mixture was boiled at 100 °C for 10–15 min to inactivate the enzyme. The mixture was then cooled to room temperature and centrifuged at 1800× *g* for 20 min at 4 °C to separate the insoluble residues. The supernatant was filtered through a 0.45 µm microporous membrane to obtain the FMH, which was subsequently freeze-dried for further analysis and application.

### 2.3. Structural Characterization of FMH

#### 2.3.1. Molecular Weight Distribution Determination

FMH was dissolved in ultrapure water to prepare a 10 mg/mL clear solution. The relative molecular weight distribution was determined by high-performance liquid chromatography (HPLC) using a Waters 1525EF system (Waters Corporation, Milford, MA, USA) equipped with a TSK gel G2000 SWXL column (7.8 mm × 300 mm) with a mobile phase of acetonitrile/water/trifluoroacetic acid (40:60:0.1, *v*/*v*/*v*). The sample was filtered through a 0.45 μm membrane, and 10 μL aliquots were injected at a flow rate of 0.5 mL/min [21].

#### 2.3.2. Analysis of Amino Acid Composition

A total of 100.0 mg of FMH was accurately weighed and transferred into a hydrolysis tube containing 8 mL of 6 mol/L HCl. After nitrogen purging for 3 min to remove oxygen, the tube was sealed and hydrolyzed at 120 °C for 22 h. The hydrolysate was cooled, transferred to a 25 mL volumetric flask, and neutralized with 4.80 mL of 10 mol/L NaOH, followed by ultrapure water dilution to volume. The solution was filtered through a 0.45 μm membrane, and 1 mL of the filtrate was centrifuged at 6750× *g* for 10 min. Subsequently, 400 μL of the supernatant was analyzed using an Agilent 1260 liquid chromatography system (Agilent Technologies, Inc., Santa Clara, CA, USA).

#### 2.3.3. FTIR Spectral Analysis

FTIR spectroscopic analysis of FMH was conducted on an iS10 Fourier-transform infrared spectrometer (Thermo Fisher Scientific Inc., Waltham, MA, USA). Lyophilized samples were directly deposited onto the ATR crystal, and spectral data were collected over the 4000–600 cm^−1^ range with 32 cumulative scans at 4 cm^−1^ resolution.

#### 2.3.4. Mass Spectrometry Identification of FMH

Nano liquid chromatography-tandem mass spectrometry (nanoLC-MS/MS) analysis was performed on an Orbitrap Exploris 480 mass spectrometer (Thermo Fisher Scientific, Bremen, Germany) coupled with a Dionex UltiMate 3000 nano HPLC system (Thermo Fisher Scientific, Waltham, MA, USA). Mass spectra were acquired in positive ion mode under data-dependent acquisition (DDA) settings: a full-scan Orbitrap MS survey followed by high-energy collisional dissociation (HCD) fragmentation of the most intense ions detected in the MS scan, with Orbitrap detection at a 2-s duty cycle.

Mass spectrometry data files were processed using Proteome Discoverer 2.4.0.305 software with the MSPepSearch and SequestHT search engines. Database searches against the UniProt/Swiss-Prot database for *Perca fluviatilis* species were conducted with the following parameters: trypsin digestion (maximum two missed cleavage sites); precursor and fragment mass tolerances set to 10 ppm and 0.05 Da, respectively; dynamic modifications including methionine oxidation and N-terminal acetylation; static modification of cysteine carbamidomethylation. Peptide identifications were filtered at a high-confidence level (false discovery rate, FDR < 1%).

For unassigned low-molecular-weight peptides, the raw MS/MS data were converted to mzXML format using MSConvert and subjected to molecular networking analysis via the Classical Molecular Networking workflow on the Global Natural Products Social Molecular Networking (GNPS, http://gnps.ucsd.edu) platform. Spectral similarity clustering was conducted with the following parameters: Precursor Ion Mass Tolerance: 0.2 Da, Fragment Ion Mass Tolerance: 0.02 Da, Cosine Score: 0.6, Library Search Min Matched Peaks: 6, and maximum analog search mass difference = 100.0 Da. The results were visualized using Cytoscape v3.8.2 (The Cytoscape Consortium, San Diego, CA, USA). The molecular network filtering parameter was set at a precursor mass < 400 Da.

### 2.4. Animals and Treatments

32 male C57BL/6J mice, 6 weeks old, were purchased from Beijing Weitong Lihua Experimental Animal Technology Co., Ltd., Beijing, China, with the license number SYXK (SU) 2021-0056. All animal experiments were approved by the Experimental Animal Welfare and Ethics Review Committee of Jiangnan University on 15 November 2023, with the approval number JN.No20231115c0900104[537]. The mice were raised in an SPF-level barrier environment, with a controlled temperature of 23–27 °C and a relative humidity of 40–60%, under an artificial light/dark cycle for 12 h.

After a one-week adaptation period, mice were randomly assigned to four groups: the normal control group (CON, n = 8), the CTX-induced model group (CTX, n = 8), the FMH-treated control group (CON+FMH, n = 8), and the CTX combined with FMH-treatment group (CTX+FMH, n = 8).

The intervention began on day 8. Mice in the CON and CTX groups were gavaged with 200 µL of normal saline daily, while those in the CON+FMH and CTX+FMH groups received 200 µL of FMH (1.67 g/kg·bw) via gavage daily for 12 days. Body weight (BW) and food intake were recorded every 3 days. On day 17, modeling was initiated by intraperitoneal injection of 200 µL cyclophosphamide (80 mg/kg, diluted in normal saline) daily for 3 days in the CTX and CTX+FMH groups. The dose of cyclophosphamide was determined based on the method described by Cheng [22]. FMH was dissolved in normal saline and administered by oral gavage. The dose of FMH (1.67 g/kg bw) was selected based on a previous study and our preliminary experiments [22]. On day 20, mice were fasted overnight with free access to water, weighed, and then anesthetized with isoflurane inhalation. Euthanasia was performed by cervical dislocation, followed by immediate dissection. Organ samples were collected on ice, weighed, and plasma, serum, and tissues were stored at −80 °C for further analysis.

### 2.5. Determination of Whole Blood Hemogram Indexes

50 µL of blood was collected from the orbital sinuses of mice into heparinized tubes for the analysis of basic hematological parameters. An automated hematology analyzer (BC-5000Vet, Shenzhen Mindray Bio-Medical Electronics Co., Ltd., Shenzhen, China) was used to measure the counts of white blood cells (WBC), red blood cells (RBC), as well as the proportions of neutrophils (Neu), eosinophils (Eos), basophils (Bas), monocytes (Mon), and lymphocytes (Lym).

### 2.6. Histopathological Examination

After euthanasia, the spleen was removed and immediately fixed in 10% neutral buffered formalin. The tissue samples were then processed through graded ethanol dehydration, cleared with xylene, and embedded in paraffin wax. The paraffin-embedded tissue blocks were processed at 65–70 °C. Sections of 4–5 µm thickness were cut and baked at 60 °C for 30 min to remove residual moisture. Following sectioning, the slides were stained with hematoxylin and eosin (H&E) for histological evaluation. The stained sections were examined and analyzed under a light microscope (NIKON ECLIPSE E100, Nikon Corporation, Tokyo, Japan).

### 2.7. Determination of Splenic Lymphocyte Proliferation Ability

Splenic lymphocyte suspensions were prepared in a procedure consistent with Li et al. [23]. In brief, a portion of the mouse spleen was collected in a sterile environment to prepare a splenic lymphocyte suspension. The proliferative capacity of mouse spleen lymphocytes induced by concanavalin A (ConA) was evaluated using the Cell Counting Kit-8 (CCK-8) assay. Lymphocytes were inoculated into 96-well plates with a total volume of 210 µL per well. The blank control group received 210 µL of RPMI-1640 complete medium, the control group received splenic lymphocyte suspension without ConA, while the ConA group received splenic lymphocyte suspension with ConA. After incubation for 68 h at 37 °C in a 5% CO_2_ incubator, 10 µL of CCK-8 solution was added to each well, followed by an additional 4 h of incubation. The optical density (OD) value of each well was measured with an enzyme-labeled instrument (BioTek Instruments, Winooski, VT, USA) at 450 nm, and the stimulation index (SI) was used as an evaluation index to reflect the proliferation ability of lymphocytes [24]. The stimulation index was calculated according to Formula (1), as follows:

(1) $$\mathrm{SI}\left(\%\right)=\frac{{\mathrm{OD}}_{\mathrm{sample}}-{\mathrm{OD}}_{\mathrm{blank}}}{{\mathrm{OD}}_{control}-{\mathrm{OD}}_{\mathrm{blank}}}\times 100$$

### 2.8. Determination of Cytokines in Serum

The levels of IL-3, IL-6, and TNF-α in serum were determined using an enzyme-linked immunosorbent assay (ELISA) kit (Huijia Biotechnology Co., Ltd., Xiamen, China). The enzyme-labeled reagents were added to 96-well plates according to the manufacturer’s instructions, followed by incubation and washing steps. After the color development reaction, the reaction was terminated by adding 1.0 M H_2_SO_4_ solution. The absorbance of each well was then measured at a wavelength of 450 nm, and the concentrations of the cytokines were calculated based on the standard curve equation.

### 2.9. Thymus Transcriptome Sequencing and Analysis

#### 2.9.1. RNA Extraction, Library Construction, and Quality Control

Total RNA was extracted from mouse thymus tissues, followed by removal of rRNA to enrich for mRNA. The enriched mRNA was reverse-transcribed into double-stranded cDNA. After end repair and A-tailing of the cDNA, adapters were ligated, and the library was amplified by PCR to construct the sequencing library.

Specifically, mRNA was captured using mRNA Capture Beads. After purification, the mRNA was fragmented at elevated temperatures. Using the fragmented mRNA as a template, the first strand of cDNA was synthesized in a reverse transcription mixture. The second strand of cDNA was synthesized concurrently with end repair and A-tailing. Adapters were then ligated to the cDNA fragments. The ligation products were purified using Hieff NGS^®^ DNA Selection Beads to select target fragments, followed by PCR amplification to complete the library construction. The quality of the constructed libraries was assessed using agarose gel electrophoresis, NanoPhotometer spectrophotometry (Implen, Munich, Germany), Qubit 2.0 Fluorometer (Thermo Fisher Scientific, Waltham, MA, USA), and Agilent 2100 Bioanalyzer (Agilent Technologies, Santa Clara, CA, USA). Sequencing was subsequently performed on the Illumina Novaseq X Plus platform (Illumina, San Diego, CA, USA).

#### 2.9.2. Differentially Expressed Genes (DEGs) Analysis

Thymus tissues were collected from three experimental groups: CON, CTX, and CTX+FMH, with three biological replicates per group. Raw reads were filtered using fastp (v0.18.0) to obtain clean reads, and rRNA reads were removed by alignment to the rRNA database using Bowtie2 (v2.2.8). The resulting clean reads were then aligned to the reference genome using HISAT2 (v2.1.0). Transcript assembly was performed using StringTie (v1.3.1), and gene expression levels were quantified by RSEM (v1.3.3). DESeq2 (v1.24.0) was utilized to identify DEGs with |log_2_FoldChange| > 1 and *p* < 0.05. Furthermore, KEGG pathway enrichment analysis was performed using the KEGG Pathway database and a hypergeometric test, as implemented on the Gene Denovo analysis platform. Pathways with Q value ≤ 0.05 were considered significantly enriched.

### 2.10. Real-Time Quantitative PCR

Total RNA was extracted from thymus tissues using the TRIzol method and reverse-transcribed into cDNA with the HiScript^®^ III RT SuperMix kit (Vazyme Biotechnology Co., Ltd., Nanjing, China) according to the manufacturer’s instructions. Quantitative real-time PCR (qRT-PCR) was performed in triplicate using the 2× Taq Pro Universal SYBR qPCR Master Mix (Vazyme Biotechnology Co., Ltd., Nanjing, China) on a real-time quantitative PCR instrument (Monad, Suzhou, China). The amplification program was as follows: initial denaturation at 95 °C for 30 s, followed by 40 cycles of 95 °C for 5 s, 60 °C for 30 s, and 72 °C for 30 s. The β-actin gene served as the endogenous reference for normalization, and relative gene expression levels were calculated using the 2^−ΔΔCt^ method. The primer sequences were obtained from previously published studies and are listed in Table 1 [25,26,27,28,29].

### 2.11. Immunohistochemical Analysis

Immunohistochemical staining was performed on paraffin-embedded tissue sections. Sections were deparaffinized in a series of dewaxing solutions (10 min each) and rehydrated through graded ethanol solutions (5 min each) to distilled water. Antigen retrieval was conducted using citrate buffer (pH 6.0) in a microwave for 15 min, followed by cooling and washing in PBS (pH 7.4). Endogenous peroxidase activity was blocked with 3% hydrogen peroxide for 25 min at room temperature, followed by washing in PBS. Sections were blocked with 3% BSA for 30 min at room temperature. Primary antibodies against PI3K (1:200 dilution) and P65 (1:200 dilution) were applied overnight at 4 °C. Sections were then incubated with HRP-conjugated secondary antibodies for 50 min at room temperature. DAB substrate was applied until a brownish-yellow color appeared, indicating positive staining. Nuclei were counterstained with hematoxylin, and sections were dehydrated through graded ethanol solutions and cleared in xylene before mounting [30,31,32]. Slides were examined under an optical microscope, and the average optical density of positive signals was analyzed using ImageJ 1.54p software.

### 2.12. Statistical Analysis

Statistical analyses were performed using GraphPad Prism 8.0.2 software. All results are expressed as mean ± standard deviation (SD). Significant differences among groups were assessed by one-way analysis of variance (ANOVA), with a significance level set at *p* < 0.05.

## 3. Results

### 3.1. Structural Characterization Results of FMH

The molecular weight (Mw) distribution of FMH was determined by HPLC (Table 2). FMH is composed of peptides with molecular weights below 1755 Da, with the majority (83.89%) distributed within the 73–580 Da range, while peptides above 1111 Da accounted for only 3.43%.

**Figure 1:**
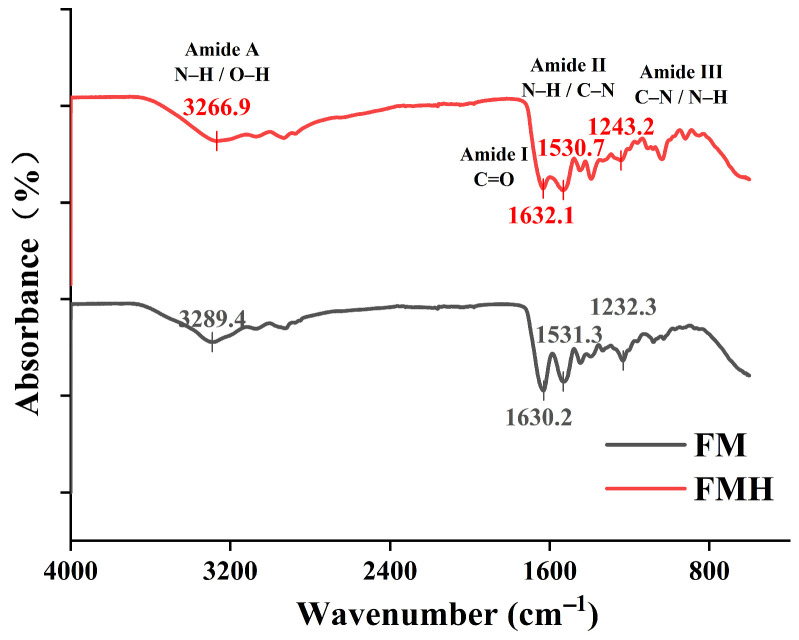
Fourier transform infrared (FTIR) spectra of FMH.

FTIR spectroscopy analysis was performed on components during FMH preparation (Figure 1). Both spectra displayed characteristic peaks of type I collagen, particularly in the amide A, I, II and III regions. The amide A band appeared at 3289.4 cm^−1^ for FM and 3266.9 cm^−1^ for FMH, mainly associated with N-H and O-H stretching vibrations. Compared with FM, FMH showed a redshift of 22.5 cm^−1^, indicating a change in the hydrogen-bonding interactions [33]. The amide I band (1700–1600 cm^−1^), dominated by C=O stretching vibrations, showed peaks at 1630.2 cm^−1^ (FM) and 1632.1 cm^−1^ (FMH) [34]. The amide II band showed peaks at 1531.3 cm^−1^ (FM) and 1530.7 cm^−1^ (FMH), mainly arising from N-H bending coupled with C-N stretching vibrations. In the amide III region (1400–1200 cm^−1^), FM and FMH exhibited bands at 1232.3 and 1243.2 cm^−1^, which are commonly used as indicators of collagen-related structural features [35]. Overall, the presence of these characteristic amide bands in FMH is consistent with the retention of collagen-derived chemical features after hydrolysis, although slight shifts in peak positions suggest changes in the local molecular environment and intermolecular interactions.

As shown in Table 3, FMH is enriched in charged amino acids (e.g., Asp, Glu, Arg, Lys, and His) and hydrophobic amino acids (e.g., Gly, Ala, Tyr, Val, Met, Phe, Ile, Leu, and Pro), which account for 28.61% and 67.33% of the total amino acid content, respectively.

High-resolution mass spectrometry (HRMS) was employed to characterize the peptide composition of FMH. Low-molecular-weight peptides (precursor mass < 400 Da) were identified through spectral matching using the GNPS online database, while larger polypeptides were further characterized by database searching against the UniProt/Swiss-Prot database of *Perca fluviatilis*. The molecular network generated 1032 nodes, with each node representing a precursor ion feature (Figure S1). Cross-referencing with the GNPS spectral library enabled the identification of five small peptides, including Asp-Leu, Gly-Pro-Ala, Glu-Val-Phe, pyroGlu-Tyr, and pyroGlu-Phe. In addition, seven polypeptides with high signal intensity were identified (relative intensity > 10^7^, Table 4), which were mainly assigned to collagen alpha-1(I), alpha-1(XII), and alpha-1(XXVIII) chains. These peptides exhibited molecular weights below 1500 Da, contained characteristic collagen-like Gly-X-Y repetitive motifs, and were enriched in amino acid residues including Gly (G), Pro (P), Ala (A), Arg (R), and Glu (E) [36].

### 3.2. FMH Ameliorates Systemic Immune Function

**Figure 2:**
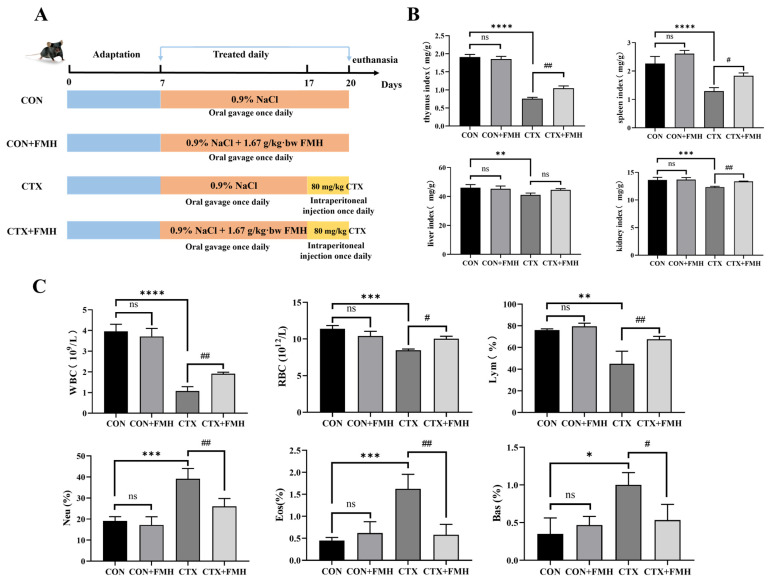
Effect of FMH on CTX-induced immunosuppressed model. (**A**) Schematic of the experimental design. (**B**) Organ indices. (**C**) peripheral blood count. Data are expressed as the mean ± SD. Compared with the CON group: ns, *p ≥* 0.05; * *p* < 0.05, ** *p* < 0.01, *** *p* < 0.001, **** *p* < 0.0001; Compared with the CTX group: ns, *p ≥* 0.05; # *p* < 0.05, ## *p* < 0.01.

A total of 32 male C57BL/6J mice were used to investigate whether FMH could alleviate immunosuppression (Figure 2A). Body weight and food intake of mice in each group were monitored during the feeding period (Figure S2). After CTX injection, both body weight gain and food intake decreased in the CTX and CTX+FMH groups, with larger decreases in the CTX group. These findings suggest that FMH may partially alleviate the CTX-induced reductions in body weight and food intake in immunosuppressed mice.

At the end of the intervention, organ indices (thymus, spleen, liver, and kidney) were evaluated across all groups (Figure 2B). Mice treated with CTX exhibited significant decreases in thymus, spleen, liver, and kidney indices compared with the CON group (*p* < 0.01). Notably, FMH intervention significantly improved the thymus, spleen, and kidney indices in CTX-treated mice (*p* < 0.05). No significant differences in organ indices were observed between the CON group and the CON+FMH group (*p* *≥* 0.05), indicating that FMH administration under physiological conditions induced no adverse effects.

Blood cell counts for each group of mice were measured (Figure 2C). A 3-day continuous intraperitoneal injection of CTX significantly reduced the levels of WBC, RBC, and Lym in the peripheral blood of mice (*p* < 0.01). Neu, Eos, and Bas increased significantly (*p* < 0.05). In contrast, no significant differences in blood cell counts were observed between the CON and CON+FMH groups (*p* *≥* 0.05), suggesting that FMH did not markedly affect peripheral blood cell profiles in normal mice. Notably, when compared with the CTX group, the CTX+FMH group exhibited significant improvement in WBC, RBC, Lym, Neu, Eos and Bas counts (*p* < 0.05).

The experimental findings suggested that FMH was well tolerated under physiological conditions and improved the thymus, spleen, and kidney indices, partially restored several peripheral blood cell parameters, and alleviated CTX-induced systemic immunosuppression.

### 3.3. FMH Enhances Splenic Immune Activity

**Figure 3:**
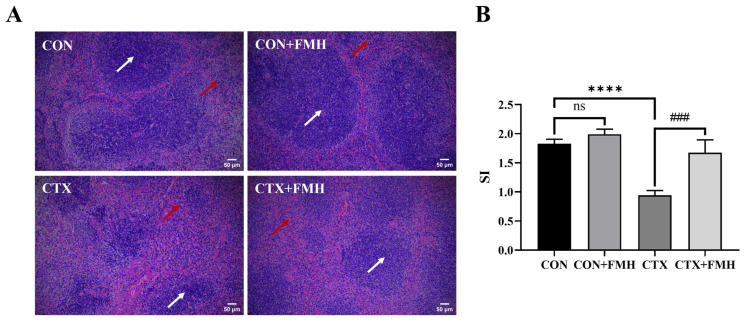
Effect of FMH on splenic immune activity. (**A**) Histological architecture of the spleen in each group (H&E staining, ×100). White arrows demarcate the white pulp regions, while red arrows highlight the red pulp areas. (**B**) Splenic lymphocyte proliferation in mice. Stimulation index (SI) was quantified to assess mitogen-induced proliferative activity. Data are expressed as the mean ± SD. Compared with the CON group: ns, *p ≥* 0.05; **** *p* < 0.0001; Compared with the CTX group: ### *p* < 0.001.

Histopathological images of H&E-stained splenic tissues are illustrated in Figure 3A. In the CON and CON+FMH groups, the splenic tissues exhibited an intact capsule, clear demarcation between red pulp and white pulp, and lymphocytes arranged in orderly patterns. In CTX-treated mice, the boundary between red pulp and white pulp was blurred, the white pulp structure was reduced, lymphocyte count decreased, cells were loosely arranged, and staining intensity was diminished, indicating that CTX caused significant structural damage to the spleen and induced immunosuppression. Compared with the CTX group, the CTX+FMH group showed distinct demarcation between red and white pulp, increased white pulp structure, and a mitigated reduction in lymphocyte count, demonstrating that FMH significantly alleviated splenic lesions in CTX-induced immunosuppressed mice. Compared with the CON group, the CTX group exhibited a significantly decreased stimulation index (SI). Notably, the SI value in the CTX+FMH group was significantly increased compared to the CTX group (*p* < 0.001), demonstrating that FMH enhances the proliferative capacity of splenic lymphocytes in CTX-induced immunosuppressed mice (Figure 3B).

### 3.4. FMH Regulates Cytokine Homeostasis

**Figure 4:**
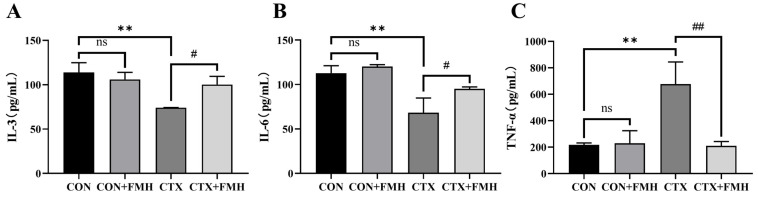
Effect of FMH on serum cytokine levels. (**A**) IL-3. (**B**) IL-6. (**C**) TNF-α. Data are expressed as the mean ± SD. Compared with the CON group: ns, *p ≥* 0.05; ** *p* < 0.01; Compared with the CTX group: # *p* < 0.05, ## *p* < 0.01.

ELISA revealed no significant differences (*p* *≥* 0.05) in serum cytokine levels between the CON and CON+FMH groups (Figure 4). Compared to the CON group, the CTX group exhibited significantly decreased IL-3 and IL-6 levels (*p* < 0.01) and increased TNF-α levels (*p* < 0.01). Relative to the CTX group, the CTX+FMH group showed significantly increased IL-3 and IL-6 levels (*p* < 0.05) and decreased TNF-α levels (*p* < 0.01), indicating that FMH effectively regulates cytokine levels.

### 3.5. Transcriptome Reveals FMH-Mediated Immunomodulation via NF-κB Pathway

#### 3.5.1. Inter-Sample Relationship and Differentially Expressed Genes (DEGs) Analysis

**Figure 5:**
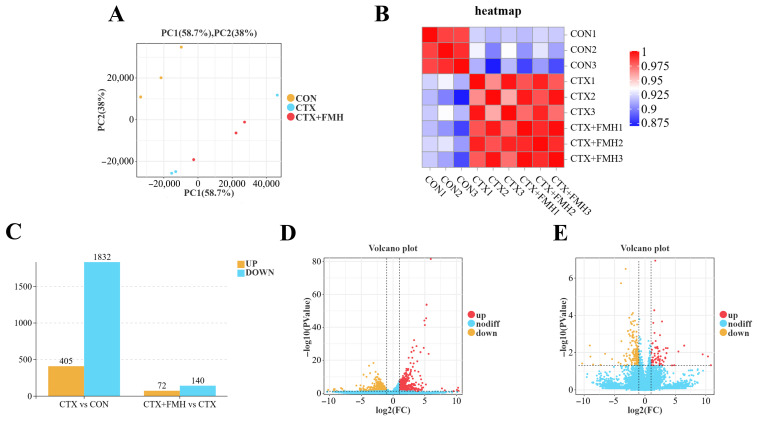
Thymus transcriptome analysis. (**A**) Principal component analysis (PCA). (**B**) Sample correlation heatmap based on Pearson correlation coefficients. (**C**) Counts of up-and downregulated differentially expressed genes (DEGs). (**D**) Volcano plot: CTX vs. CON. (**E**) Volcano plot: CTX+FMH vs. CTX.

The PCA scatter plot revealed a clear grouping trend among the different groups (Figure 5A). Specifically, the data points for the CTX and CTX+FMH groups showed some overlap along the first principal component (PC1) but were more dispersed along the second principal component (PC2). In contrast, the CON group data points were distinctly separated from those of the CTX and CTX+FMH groups along PC1, and they also exhibited significant differences along PC2. This distribution pattern suggests that the CTX and CTX+FMH groups showed partial similarity in overall transcriptomic profiles, while still exhibiting separation along the second principal component. Although one sample showed a relatively distinct distribution in the PCA plot, this may reflect biological variability among individual mice. Nevertheless, the overall grouping trend remained clear, indicating that CTX markedly altered thymus transcriptomic profiles.

To further validate the correlations between samples, we generated a heatmap based on Pearson correlation coefficients to visualize sample-to-sample correlations (Figure 5B). The correlation coefficients between all samples were above 0.875, indicating good inter-sample consistency. This provides assurance of data quality for subsequent differential gene expression analysis. In the CTX vs. CON group, a total of 2237 DEGs were identified, with 405 upregulated genes and 1832 downregulated genes. In the CTX+FMH vs. CTX group, a total of 212 DEGs were identified, with 72 upregulated genes and 140 downregulated genes (Figure 5C–E). The full lists of identified genes and DEGs for these two comparisons are provided in Supplementary Tables S1–S4.

#### 3.5.2. KEGG Enrichment Analysis

**Figure 6:**
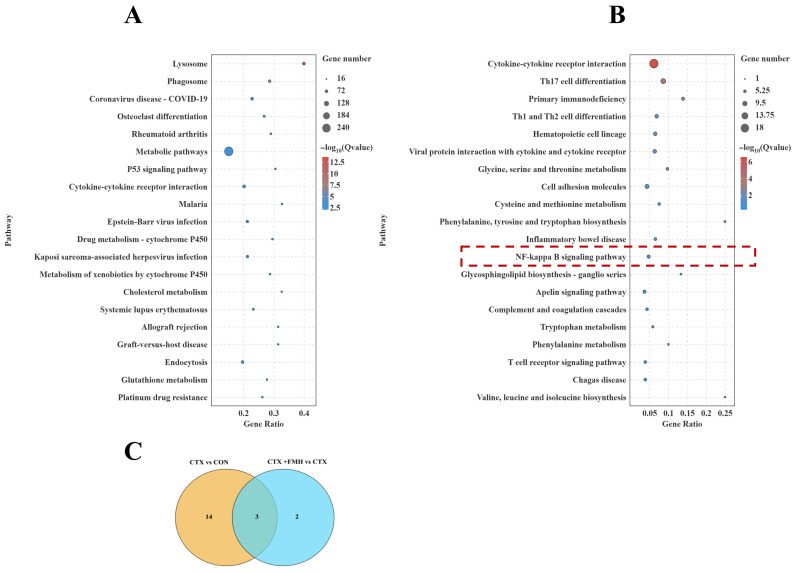
KEGG pathway enrichment bubble plots of DEGs (bubble size: gene count; color: −log10(Q value)). (**A**) CTX vs. CON. (**B**) CTX+FMH vs. CTX; the dotted red box highlights the NF-κB signaling pathway selected for further analysis. (**C**) Venn diagram of DEGs enriched in the NF-κB signaling pathway.

KEGG enrichment analysis was performed on the DEGs identified in each comparison (Figure 6). In the CTX vs. CON comparison, the top enriched pathways included lysosome, phagosome, coronavirus disease-COVID-19, osteoclast differentiation, rheumatoid arthritis, metabolic pathways, p53 signaling pathway, and cytokine-cytokine receptor interaction, suggesting that CTX induced broad transcriptomic alterations associated with immune regulation, stress responses, and metabolic disturbance. In the CTX+FMH vs. CTX comparison, the enriched pathways were mainly associated with cytokine-cytokine receptor interaction, Th17 cell differentiation, primary immunodeficiency, Th1 and Th2 cell differentiation, hematopoietic cell lineage, viral protein interaction with cytokine and cytokine receptor, inflammatory bowel disease, T cell receptor signaling pathway, and NF-κB signaling pathway. These results suggest that FMH exerts its protective effect against CTX-induced immunosuppression mainly through modulation of immune-related signaling networks.

Notably, the NF-κB signaling pathway was significantly enriched in the CTX+FMH vs. CTX comparison. As NF-κB is a central regulator of innate and adaptive immune responses and controls the transcription of multiple inflammatory mediators, this pathway was considered particularly relevant to the immunological focus of the present study and was therefore selected for further mechanistic validation [37]. The Venn diagram showed that three NF-κB pathway-related DEGs overlapped between the two comparisons, namely *IL-1β*, *Ltb*, and *Zap70*. Among them, *IL-1β* was upregulated in the CTX vs. CON comparison but downregulated in the CTX+FMH vs. CTX comparison, whereas *Ltb* and *Zap70* showed the opposite trend, being downregulated in the CTX vs. CON comparison but upregulated in the CTX+FMH vs. CTX comparison (Figure 6C).

### 3.6. Mechanistic Validation of FMH-Mediated NF-κB Pathway Modulation

#### 3.6.1. Transcriptional Profiling via Quantitative Real-Time PCR (qRT-PCR)

**Figure 7:**
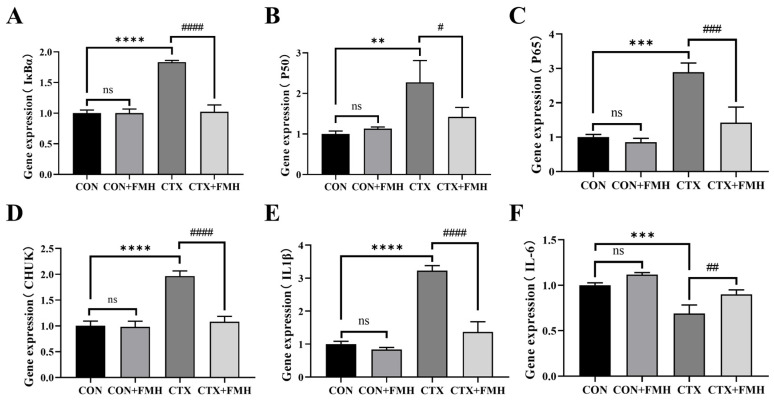
Effect of FMH on the expression of NF-κB pathway-related genes in CTX-induced immunosuppressed mice. (**A**) *IκBα*, (**B**) *P50*, (**C**) *P65*, (**D**) *CHUK*, (**E**) *IL-1β*, (**F**) *IL-6*. Data are expressed as mean ± SD. Compared with the CON group: ns, *p ≥* 0.05; ** *p* < 0.01, *** *p* < 0.001, **** *p* < 0.0001; compared with the CTX group: # *p* < 0.05, ## *p* < 0.01, ### *p* < 0.001, #### *p* < 0.0001.

To further investigate the mechanisms by which FMH mitigates CTX-induced immunosuppression, the thymic mRNA expression levels of key NF-κB signaling pathway-related genes, including *IκBα*, *P50*, *P65, CHUK*, *IL-1β* and *IL-6*, were determined by qRT-PCR (Figure 7). Although *Ltb* and *Zap70* were also identified among the overlapping NF-κB pathway-related DEGs in the transcriptome analysis, they were not prioritized for RT-qPCR validation because they mainly act upstream of NF-κB signaling, whereas the selected genes more directly reflect the core NF-κB cascade and its downstream inflammatory outputs [38,39]. No significant differences in NF-κB-related gene expression were observed between the CON and CON+FMH groups (*p* *≥* 0.05). In contrast, compared with the CON group, CTX treatment significantly increased the expression levels of *IκBα*, *P50*, *P65*, *CHUK*, and *IL-1β* (*p* < 0.01), alongside marked downregulation of *IL-6* (*p* < 0.001). Notably, compared with the CTX group, FMH treatment significantly reversed the CTX-induced changes in the expression of *IκBα*, *P50*, *P65*, *CHUK*, *IL-1β*, and *IL-6* (*p* < 0.05), suggesting that FMH may alleviate CTX-induced immunosuppression by modulating NF-κB signaling and its downstream inflammatory responses, which is consistent with the transcriptomic findings.

#### 3.6.2. Validation Through Immunohistochemical (IHC) Staining

**Figure 8:**
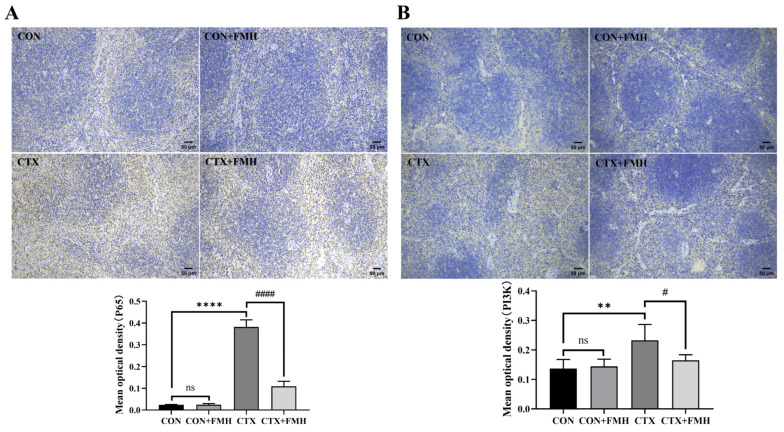
Immunohistochemical staining (IHC, ×100) of P65 and PI3K in splenic tissues from each group. (**A**) NF-κB P65, (**B**) PI3K. Data are expressed as mean ± SD. Compared with the CON group: ns, *p ≥* 0.05; ** *p* < 0.01, **** *p* < 0.0001; compared with the CTX group: *# p* < 0.05, #### *p* < 0.0001.

Immunohistochemical (IHC) staining was performed to evaluate the protein expression of P65 and PI3K in splenic tissues (Figure 8). No significant differences in P65 or PI3K expression were observed between the CON and CON+FMH groups (*p* *≥* 0.05). Compared with the CON group, the CTX group showed significantly increased expression of both P65 and PI3K (*p* < 0.01). In contrast, FMH significantly reduced the expression levels of these proteins compared with the CTX group (*p* < 0.05).

## 4. Discussion

The immune system protects the host against pathogenic invasions and maintains physiological homeostasis [40]. Recently, diet-derived bioactive immunomodulators have attracted significant interest due to their natural origin and functional efficacy [41]. Studies indicate that bioactive peptides from natural products, such as soybean [42] and *Mytilus coruscus* [43] exhibit immunomodulatory activity through regulation of immune cell function and cytokine secretion. While extensive investigations have been conducted on the immunomodulatory functions of bioactive peptides from diverse biological sources, the immunomodulatory properties and mechanisms of peptides in fish maw hydrolysate (FMH) remain unclear. In the present study, FMH derived from freshwater perch (*Perca fluviatilis*) maw exhibited immunomodulatory activity in CTX-immunosuppressed mice. FMH consisted mainly of low-molecular-weight peptides and was enriched in charged and hydrophobic amino acids. Moreover, FMH ameliorated CTX-induced alterations in immune organ indices, peripheral blood cell parameters, and cytokine profiles, possibly through modulation of the NF-κB signaling pathway. These findings expand the potential raw material sources for fish maw product development and support the development of dietary immunomodulators.

The immunomodulatory activity of bioactive peptides is linked to their structural characteristics, including relative molecular mass, amino acid composition, sequence arrangement, chain length, and hydrophobicity [44]. The FTIR spectrum of FMH exhibited characteristic peaks of type I collagen, indicating the presence of collagen-derived chemical features after hydrolysis. Low-molecular-weight peptides are often associated with enhanced immunomodulatory activity [45]. FMH contains peptides with molecular weights below 1755 Da, with the majority (83.89%) distributed within 73–580 Da. Consequently, the potential bioactivity of FMH may be partly attributed to the short-chain peptides generated during hydrolysis. In addition, HRMS analysis identified multiple small peptides and collagen-derived peptide fragments in FMH, further supporting the compositional complexity of the low-molecular-weight peptide fraction that may contribute to its bioactivity. Previous studies have reported that hydrophobic amino acids enhance immunomodulatory activity by strengthening peptide-membrane interactions [46]. Charged amino acids may also contribute to immunomodulatory effects through interactions with immune cells or related receptors [47,48]. FMH is enriched in charged amino acids and hydrophobic amino acids, which account for 28.61% and 67.33% of the total amino acid content, respectively. This compositional profile may contribute to its potential immunomodulatory activity.

CTX, an alkylating cytotoxic agent, is widely used to induce immunosuppression in experimental models [19]. Mechanistically, CTX can cause oxidative DNA damage and impair the proliferation and function of immune cells, thereby inducing immune dysfunction [49,50]. Compared with the CON group, CTX significantly reduced the organ indices of mice (spleen, thymus, liver, and kidney indices; *p* < 0.01). The decreases in WBCs, RBCs, and lymphocytes further indicated that immune function was significantly suppressed in CTX-treated mice [51,52]. H&E staining revealed structural lesions in splenic tissues characterized by white pulp atrophy and disordered lymphocyte arrangement. Furthermore, CTX significantly suppressed the SI of splenic lymphocytes (*p* < 0.0001) and concurrently decreased serum levels of IL-3 and IL-6, while elevating TNF-α expression (*p* < 0.01). These findings confirm the successful establishment of an immunosuppressive murine model induced by CTX.

As pivotal immune organs, the spleen and thymus are essential for immune cell differentiation, maturation, and antigen-specific responses [53]. FMH intervention significantly reversed CTX-induced atrophy of immune organs (*p* < 0.05). Histopathological evaluation showed that the CTX+FMH group exhibited marked restoration of splenic white pulp architecture, suggesting that FMH may alleviate CTX-induced splenic injury. Additionally, the proliferative capacity of splenic lymphocytes is widely used as an indicator of immune function [54]. FMH significantly enhanced the SI in CTX-treated mice (*p* < 0.001), suggesting its potential to ameliorate CTX-induced immune impairment by restoring cellular immune function. Importantly, no significant differences in immune organ indices or SI were observed between the CON and CON+FMH groups (*p* *≥* 0.05). Therefore, FMH appears to be well tolerated under physiological conditions and may mitigate CTX-induced immunosuppression by preserving immune organ integrity and improving cellular immune function.

Peripheral blood cells are important indicators of immune status. No significant differences in peripheral blood cell profiles were observed between the CON and CON+FMH groups (*p* *≥* 0.05), suggesting that FMH was well tolerated under physiological conditions. FMH intervention ameliorated CTX-induced alterations in several peripheral blood cell parameters, indicating its potential to alleviate CTX-driven immune cell dysregulation.

Cytokines play important roles in immune regulation by modulating innate and adaptive immune responses [55,56]. Octopus-derived peptides ameliorate CTX-mediated immunosuppression by activating IL-1β, IL-6, and TNF-α secretion pathways [57]. Shark peptides achieve immune homeostasis by modulating the secretion balance of IL-4, IL-6, IL-10, and TNF-α [58]. *Cyclina sinensis* pentadecapeptide effectively counteracts CTX-induced immunotoxicity through upregulation of IL-6, IL-1β, and TNF-α expression levels [59]. In the present study, FMH significantly modulated the serum levels of IL-3, IL-6, and TNF-α in immunocompromised mice (*p* < 0.05), suggesting its involvement in the regulation of systemic immune responses.

The thymus provides a unique microenvironment essential for the differentiation and maturation of T lymphocytes [60]. To further investigate the regulatory mechanisms of FMH on CTX-induced immunosuppression, thymic tissues from mice were collected for transcriptome profiling. KEGG analysis showed CTX-induced perturbations in pathways related to transport and catabolism, cell growth and death, immune diseases, and signaling interactions, including lysosome, phagosome, p53 signaling pathway, and cytokine-cytokine receptor interaction. FMH modulated CTX-induced immunosuppression through pathways associated with cytokine signaling, T-cell differentiation, immune dysfunction, and inflammatory signal transduction, including cytokine-cytokine receptor interaction, Th17 cell differentiation, Th1 and Th2 cell differentiation, primary immunodeficiency, inflammatory bowel disease, and the NF-κB signaling pathway.

The NF-κB pathway, governing innate immunity, adaptive immunity, inflammatory stress responses, B cell development, and lymphocyte formation, can be activated through canonical and non-canonical mechanisms [61,62]. Recent studies have demonstrated that collagen peptides from cod skin alleviate colitis by suppressing the phosphorylation of NF-κB *P65*, *IκBα*, and *P38 MAPK* in the NF-κB/MAPK pathway [34], while Lee et al. reported that fish collagen peptides promote thymic regeneration in CTX-damaged mice via the NF-κB signaling pathway [63]. In the current study, transcriptomic analysis revealed significant enrichment of the NF-κB signaling pathway in the CTX+FMH vs. CTX comparison, suggesting that NF-κB signaling may be involved in the immunomodulatory effect of FMH.

qRT-PCR validation of key NF-κB pathway genes demonstrated that FMH modulated the expression of both core regulatory components and downstream targets in thymic tissues of CTX-induced immunocompromised mice. Specifically, FMH modulated the expression of *IκBα* (a member of the IκB family), *P50/P65* (NF-κB family transcription factors), and *IKKα* (*CHUK*, a catalytic subunit of the IKK complex), while also modulating downstream inflammatory mediators *IL-6* and *IL-1β*. These results align with prior findings, suggesting that FMH mitigates thymic inflammatory injury and ameliorates CTX-induced immune dysfunction through modulation of the NF-κB-related inflammatory pathway and its downstream cascades.

P65, a key component of the NF-κB pathway, undergoes nuclear translocation following dissociation from IκB, thereby activating the transcription of cytokines and chemokines [37]. PI3K activation triggers AKT-mediated phosphorylation of the IKK complex, subsequently inducing cascading regulation of the NF-κB pathway [64]. In this study, no significant differences in P65 and PI3K protein expression were observed between the CON and CON+FMH groups in splenic tissues (*p* *≥* 0.05). CTX treatment significantly upregulated the expression levels of both P65 and PI3K proteins (*p* < 0.01), whereas FMH intervention markedly attenuated their overexpression in immunosuppressed mice (*p* < 0.05). Together with the transcriptomic and qRT-PCR results obtained from thymic tissues, these findings suggest that FMH may ameliorate CTX-induced immune dysregulation through coordinated modulation of the NF-κB pathway.

## 5. Conclusions

This study demonstrates the immunomodulatory activity and a possible mechanism of FMH derived from *Perca fluviatilis* maw. Structural characterization showed that FMH, predominantly distributed within 73–580 Da (83.89%), is enriched in hydrophobic and charged amino acids and contains small peptides and collagen-derived polypeptides. Moreover, in a CTX-induced immunosuppressed mouse model, FMH alleviated CTX-induced immune suppression by partially improving immune organ indices, ameliorating alterations in peripheral blood cell parameters, and regulating cytokine balance. Mechanistically, FMH may enhance immune responses through the modulation of the NF-κB pathway and its downstream pro-inflammatory cytokine cascades. These findings provide a new strategy for the high-value utilization of freshwater fish maw and offer a basis for expanding dietary sources of immunomodulatory hydrolysate. Further studies on dose dependence, safety, and validation in other relevant models would help support its future application.

## Figures and Tables

**Table 1:** Primer sequences used for qRT-PCR.

Gene	Forward Primer (5′–3′)	Reverse Primer (5′–3′)
*β-actin*	GGCTGTATTCCCCTCCATCG	CCAGTTGGTAACAATGCCATGT
*IκBα*	GAGCTCCGAGACTTTCGAGG	AGACACGTGTGGCCATTGTA
*P50*	CTCTGGCACAGAAGTTGGGT	TCCCGGAGTTCATCTCATAGT
*P65*	GGATTCCGGGCAGTGACG	GAGGGGAAACAGATCGTCCA
*CHUK*	CCCTCCAGTATCAGCATGGC	GTGCTAACGTCTCTCACACA
*IL-1β*	TGCCACCTTTTGACAGTGATG	TGATGTGCTGCTGCGAGATT
*IL-6*	GACAAAGCCAGAGTCCTTCAGA	TGTGACTCCAGCTTATCTCTTGG

**Table 2:** Molecular weight distribution of FMH.

	Molecular Weight (Da)	Relative Content (%)
1	1111~1755	3.43
2	580~1111	11.04
3	235~580	37.64
4	73~235	46.25
5	<73	1.63

**Table 3:** Amino Acid Composition Analysis of FMH.

Amino Acids	Relative Content (%)	Amino Acids	Relative Content (%)
Asp **	5.04	Cys-s	0.20
Glu **	10.54	Val *	2.33
Ser	2.39	Met *	3.67
His **	0.38	Phe *	2.03
Gly *	27.64	Ile *	1.29
Thr	1.46	Leu *	3.36
Arg **	9.14	Lys **	3.51
Ala *	11.65	Pro *	14.56
Tyr *	0.80		

* Hydrophobic amino acids; ** Charged amino acids.

**Table 4:** Polypeptide Sequence Analysis of FMH.

	*m*/*z*	MH+ [Da]	Amino Acid Sequence	Protein Source
1	683.82	1366.63	[G].PAGERGEQGPSGAPG.[F]	Collagen alpha-1(I) chain
2	475.26	949.52	[E].AGPAGPIGPRG.[D]	Collagen alpha-1(I) chain
3	463.73	926.45	[E].PGASGPMGPR.[G]	Collagen alpha-1(XII) chain
4	626.31	626.31	[L].PGEVGAP.[G]	Collagen alpha-1(I) chain
5	497.27	497.27	[TVP].GPAGPV.[G]	Collagen alpha-1(XXVIII) chain
6	780.43	780.43	[R].GLPGVPGPS.[G]	Collagen alpha-1(I) chain
7	570.29	570.29	[NP].TGPAGAP.[G]	Collagen alpha-1(XXVIII) chain

## Data Availability

The raw transcriptomic sequencing data generated in this study have been deposited in the NCBI Sequence Read Archive (SRA) under BioProject accession number PRJNA1440437. The original contributions presented in the study are included in the article and its Supplementary Materials. Further inquiries can be directed to the corresponding authors.

## Supplementary Materials

The following supporting information can be downloaded at: https://www.mdpi.com/article/10.3390/foods15071227/s1, Figure S1: Molecular network of FMH generated by GNPS; Figure S2: Effects of FMH on body weight and food intake; Table S1: Full list of identified genes in the CTX vs. CON comparison; Table S2: Full list of identified genes in the CTX+FMH vs. CTX comparison; Table S3: Differentially expressed genes (DEGs) in the CTX vs. CON comparison; Table S4: Differentially expressed genes (DEGs) in the CTX+FMH vs. CTX comparison.
