# Supplementary material for: Intervention of Fish (Perca fluviatilis) Maw Hydrolysate in Cyclophosphamide-Induced Immunosuppressed Mice via NF-κB Pathway
Source: Foods. 2026 Apr 3;15(7):1227. doi: 10.3390/foods15071227 (PMC13072735; doi:10.3390/foods15071227)
Supplement: Supplementary file 1 [file foods-15-01227-s001.zip › Supplementary Figures.pdf]

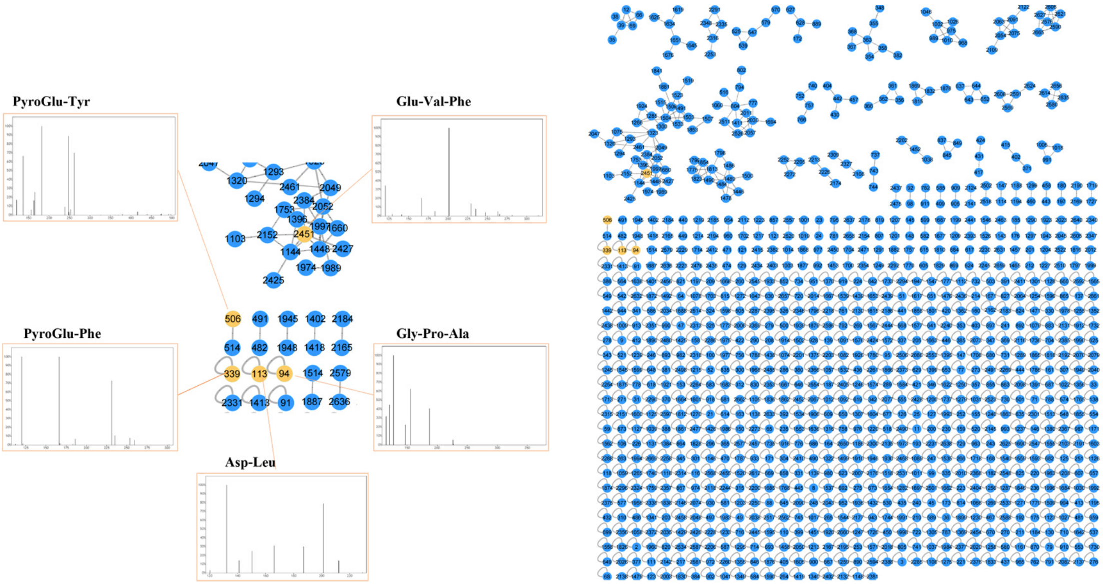

Figure S1. Molecular network of FMH generated by GNPS. Each node represents a precursor ion feature, and edges between nodes indicate spectral similarity based on cosine scores.

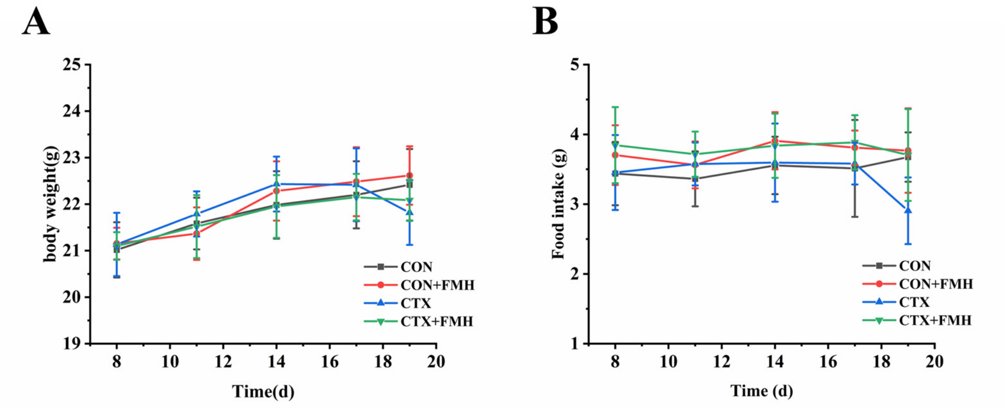

Figure S2. Effects of FMH on body weight and food intake. (A) Body weight. (B) Food intake.
